# Scrapie susceptibility-associated indel polymorphism of shadow of prion protein gene (*SPRN*) in Korean native black goats

**DOI:** 10.1038/s41598-019-51625-8

**Published:** 2019-10-24

**Authors:** Yong-Chan Kim, Seon-Kwan Kim, Byung-Hoon Jeong

**Affiliations:** 1 Korea Zoonosis Research Institute, Chonbuk National University, Iksan, 54531 Republic of Korea; 2 Department of Bioactive Material Sciences and Institute for Molecular Biology and Genetics, Chonbuk National University, Jeonju, 54896 Republic of Korea

**Keywords:** Population genetics, Genotype, Haplotypes, Genetic linkage study, Genetic association study

## Abstract

Prion diseases in sheep and goats are called scrapie and belong to a group of transmissible spongiform encephalopathies (TSEs) caused by the abnormal misfolding of the prion protein encoded by the prion protein gene (*PRNP*). The shadow of the prion protein gene (*SPRN*) is the only prion gene family member that shows a protein expression profile similar to that of the *PRNP* gene in the central nervous system. In addition, genetic susceptibility of the *SPRN* gene has been reported in variant Creutzfeldt–Jakob disease (CJD), bovine spongiform encephalopathy (BSE) and scrapie. However, genetic studies of the *SPRN* gene have not been carried out in Korean native black goats. Here, we investigated the genotype and allele frequencies of *SPRN* polymorphisms in 213 Korean native black goats and compared these polymorphisms with those previously reported for scrapie-affected animals. We found a total of 6 polymorphisms including 1 nonsynonymous single nucleotide polymorphism (SNP) and 1 synonymous SNP in the open reading frame (ORF) region and 3 SNPs and 1 indel polymorphism (c.495_496insCTCCC) in the 3′ untranslated region (UTR) by direct DNA sequencing. A significant difference in the allele frequency of the c.495_496insCTCCC indel polymorphism was found between the Italian scrapie-affected goats and the Korean native black goats (P < 0.001). Furthermore, there was a significant difference in the allele frequencies of the c.495_496insCTCCC indel polymorphism between Italian healthy goats and Korean native black goats (P < 0.001). To evaluate the biological impact of the novel nonsynonymous SNP c.416G > A (Arg139Gln), we carried out PROVEAN analysis. PROVEAN predicted the SNP as ‘Neutral’ with a score of −0.297. To the best of our knowledge, this is the first genetic study of the *SPRN* gene in Korean native black goats.

## Introduction

Prion diseases, also known as transmissible spongiform encephalopathies (TSEs), are fatal neurodegenerative disorders caused by pathogenic forms of the prion protein (PrP^Sc^), which is induced by structural changes of the normal prion protein (PrP^C^) in the central nervous system^1^. Scrapie in sheep and goats is classified as a TSEs, which affect a broad host range, such as humans and other animals^2,3^.

It is known that the susceptibility to prion diseases is affected by genetic polymorphisms of the prion protein gene (*PRNP*) that encodes the prion protein. In sheep, the *PRNP* haplotypes VRQ, ARQ and ARR at codons 136, 154 and 171 play important roles in scrapie susceptibility^4–8^. The ARQ/ARQ genotype has a highly significant association with scrapie susceptibility in Greek and Canadian sheep^5,9^. By contrast, the VRQ/VRQ genotype is correlated with the highest genetic susceptibility to scrapie, and the ARR/ARR genotype is well known for scrapie-resistance in the UK^4^. In goats, alleles 143R, 146N, 154R, 211R and 222Q in the relevant codons of the *PRNP* gene have been associated with scrapie susceptibility in Greece, France and Cyprus^10–13^. The alleles 154H, 211Q and 222K in the relevant codons of the *PRNP* gene have been found to be associated with scrapie resistance in French and Greek goats^11,12,14^. In addition, alleles 146S and 146D have also been associated with scrapie resistance^15–18^.

However, although variants of the *PRNP* gene have a strong impact on the development of prion diseases, inbred mouse lines that contain identical DNA sequences to these prion proteins had varying incubation periods for prion disease. This result implies that other factors in addition to the *PRNP* gene contribute to the pathogenesis of prion diseases^19^. The shadow of prion protein gene (*SPRN*), a member of the prion gene family, is a potential candidate that is associated with prion disease susceptibility^20–23^. In previous studies, genetic polymorphisms of the *SPRN* gene showed significant associations with the susceptibility to prion diseases^20,23–27^. In humans, frame shift mutations induced by a single base pair insertion at codon 46 of the *SPRN* gene were found in two variant CJD patients^20^. In sheep, two alanine-deletion polymorphisms located in the hydrophobic region of the Sho protein showed significantly different genetic distributions between scrapie-affected animals and healthy animal^24^. In goats, an indel polymorphism (602_606insCTCCC) in the 3′ untranslated region (UTR) was related to scrapie susceptibility^20^. In cattle, a 12 bp deletion polymorphism was observed in the hydrophobic regions of *SPRN* in one L-type atypical bovine spongiform encephalopathy (BSE)-affected domestic cow^23^. Although several case-control studies in various hosts of prion disease have reported associations between *SPRN* polymorphisms and prion disease susceptibilities, polymorphisms of the *SPRN* gene in Korean native goats have not been reported thus far. In Korea, the scrapie surveillance system examines 150 head of Korean native black goats each year and scrapie has not been reported thus far (http://www.qia.go.kr/bbs/openAdm/listInfoDataWebAction.do?type=0).

In the present study, we investigated genetic polymorphisms of the *SPRN* gene by using direct DNA sequencing and genotyping with *SPRN* gene-specific primers in 213 healthy Korean native black goats. In addition, we compared the genetic distribution of an insertion polymorphism of the *SPRN* gene in healthy Korean native black goats with that of scrapie-affected goats from previous studies. We also performed a linkage disequilibrium (LD) test on *SPRN* gene polymorphisms. Furthermore, we evaluated the biological impact of nonsynonymous single nucleotide polymorphisms (SNPs) of the *SPRN* gene using PROVEAN^28,29^.

## Results

**Figure 1:**
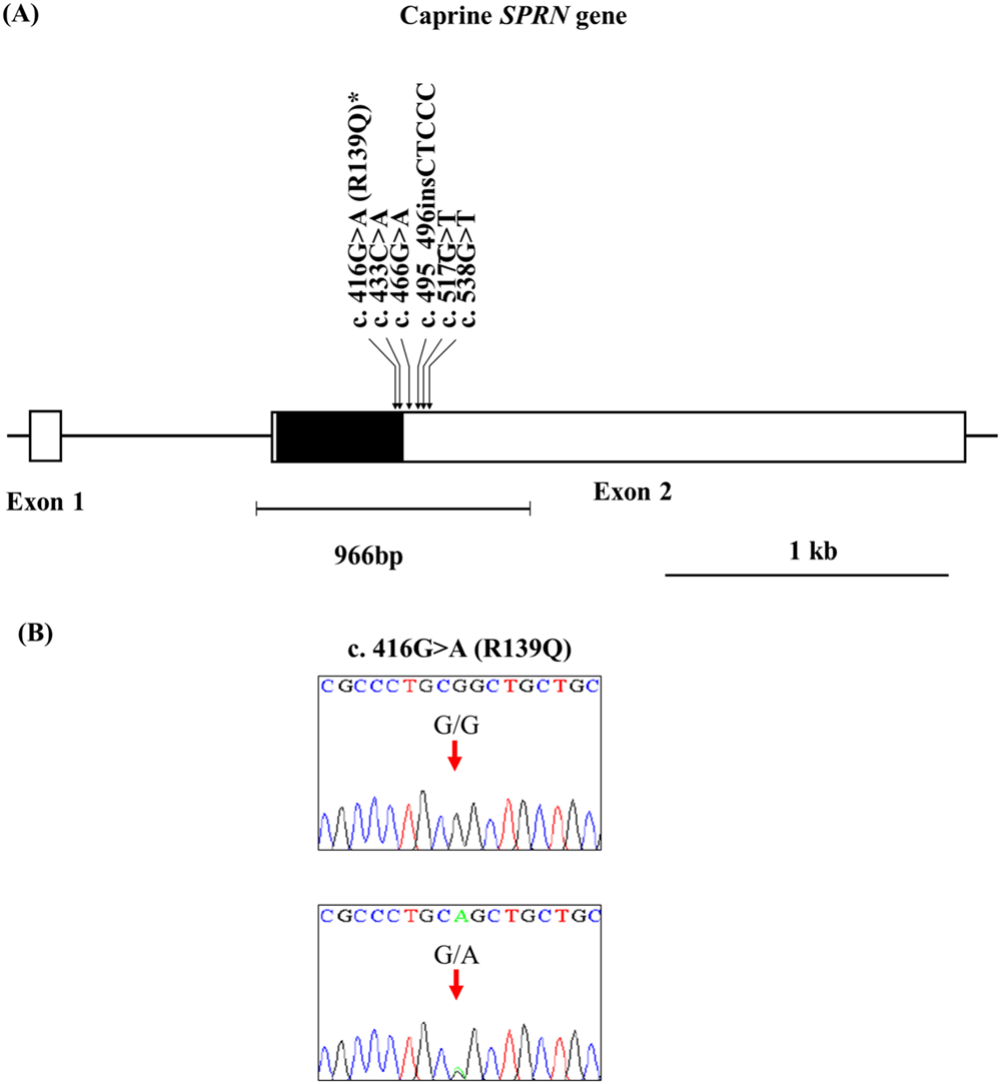
(**A**) Gene map and polymorphisms identified in the shadow of prion protein gene (*SPRN*) on chromosome 26. The open reading frame (ORF) within exon 2 is marked by a shaded block, and the 5′ and 3′ untranslated regions (UTRs) are indicated by a white block. The arrows indicate the six polymorphisms found in this study. The edged horizontal bar indicates the region sequenced. Asterisks denote novel single nucleotide polymorphisms (SNPs). (**B**) Electropherogram of novel SNPs of the *SPRN* gene. The four colors indicate individual bases of the DNA sequence using an ABI 3730 automatic sequencer (blue: cytosine; red: thymine; black: guanine; and green: adenine).

The caprine *SPRN* gene is composed of two exons. To investigate the genotype and allele frequencies of *SPRN* polymorphisms, we performed direct sequencing in the ORF region and part of the 3′UTR of the caprine *SPRN* gene in 213 Korean native black goats. We found a total of 6 polymorphisms, including c.416G > A and c.433C > A in the ORF region; c.466G > A, c.517G > T and c.538G > T in the 3′UTR; and c.495_496insCTCCC in the 3′UTR (c.495_496insCTCCC was previously described as 602_606insCTCCC^20^) (Fig. 1A). Among them, c.416G > A (R139Q) is a novel nonsynonymous SNP (Fig. 1B). Detailed values of the genotype and allele frequencies of the caprine *SPRN* gene are described in Table 1. Except for c.538G > T, all other polymorphisms were in Hardy-Weinberg equilibrium **(HWE)** proportions (Table 1).

**Table 1:** Genotype and allele frequencies of *SPRN* polymorphisms in Korean native black goats.

	Genotype frequency, n (%)			Allele frequency, n (%)		HWE
c.416G > A	GG	GA	AA	G	A	
(R139Q)	208 (97.65)	5 (2.35)	0 (0.00)	421 (98.83)	5 (1.17)	0.8624
c.433C > A	CC	CA	AA	C	A	
(R145R)	187 (87.79)	26 (12.21)	0 (0.00)	400 (93.90)	26 (6.10)	0.3428
c.466G > A	GG	GA	AA	G	A	
	123 (57.75)	82 (38.50)	8 (3.76)	328 (77.00)	98 (23.00)	0.2056
c.495_496insCTCCC	Wt/ Wt	Wt/Ins	Ins/Ins	Wt	Ins	
	202 (94.84)	11 (5.16)	0 (0.00)	415 (97.42)	11 (2.58)	0.6989
c.517G > T	GG	GT	TT	G	T	
	184 (86.38)	29 (13.62)	0 (0.00)	397 (93.19)	29 (6.81)	0.2864
c.538G > T	GG	GT	TT	G	T	
	210 (98.59)	2 (0.94)	1 (0.47)	422 (99.06)	4 (0.94)	<0.01

To examine whether there were strong LD values among the 6 polymorphisms, we investigated Lewontin’s D’ (|D’|) values. Of these, there was a weak LD between c.416G > A and c.517G > T (0.075, Table 2). Except for c.433C > A (0.564), the c.495_496insCTCCC insertion polymorphism was in strong LD with 4 SNPs, c. 416G > A, c.466G > A, c.517G > T and c.538G > T (Table 2). Detailed LD values of the caprine *SPRN* gene are described in Table 2. As shown in Table 3, 6 major haplotypes were identified in Korean native black goats. Among the 6 haplotypes, the GCGWtGG haplotype was most frequently observed (67.5%), followed by GCAWtGG (14.8%), GCAWtAG (6.8%) and GAGWtGG (6.1%, Table 3).

**Table 2:** Linkage disequilibrium (LD) of six *SPRN* polymorphisms in Korean native black goats.

	c.416G > A (R139Q)	c.433C > A (R145R)	c.466G > A	c.495_496insCTCCC	c.517G > T	c.538G > T
c.416G > A (R139Q)	—	1.0	1.0	1.0	0.075	1.0
c.433C > A (145R)	—	—	1.0	0.564	1.0	1.0
c.466G > A	—	—	—	1.0	1.0	1.0
c.495_496insCTCCC	—	—	—	—	1.0	1.0
c.517G > T	—	—	—	—	—	1.0
c.538G > T	—	—	—	—	—	—

**Table 3:** Haplotype frequencies of 6 *SPRN* polymorphisms in Korean native black goats.

Haplotype	Korean native black goats (%)
GCGWtGG	0.675
GCAWtGG	0.148
GCAWtAG	0.068
GAGWtGG	0.061
GCGInsGG	0.024
ACAWtGG	0.012
Others^a^	0.012

^a^Others contain rare haplotypes with frequency < 0.01.

**Figure 2:**
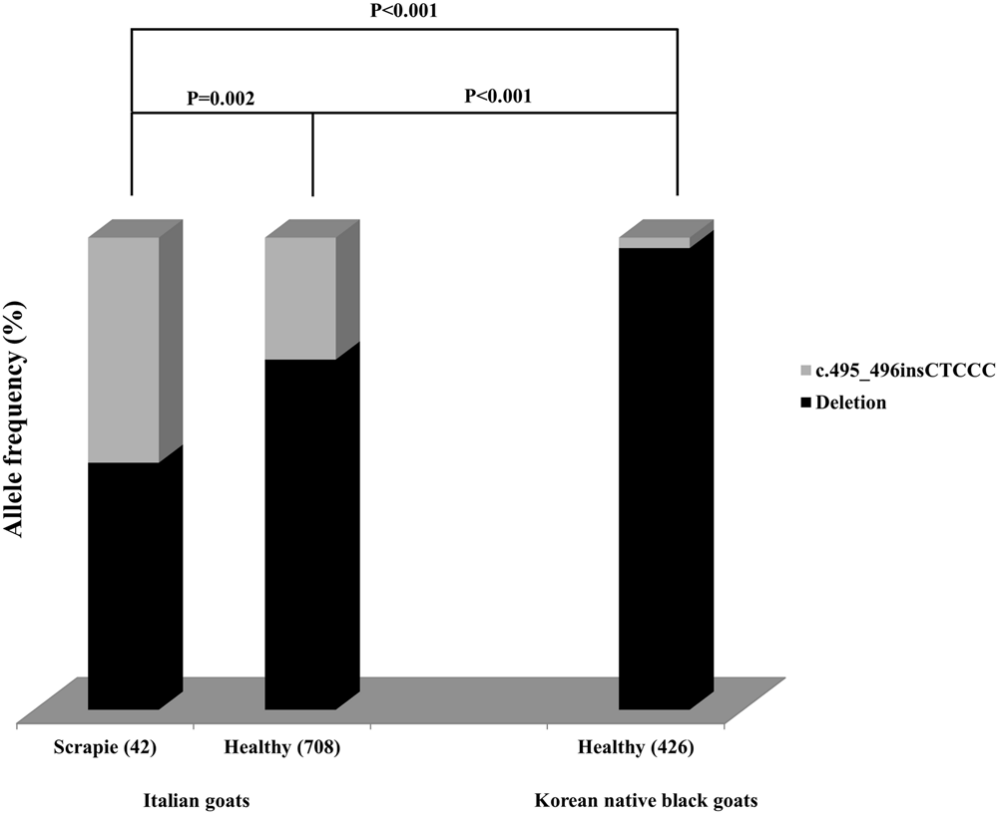
Comparison of the allele distribution of the *SPRN* c.495_496insCTCCC indel polymorphism between scrapie-affected Italian goats and Korean native black goats. The information of the Italian goats was taken from a previous study^20^. The Italian goats were Maltese, Red Mediterranean, Capra dei Nebrodi and crossed breeds. Differences in allele distribution were calculated by the Chi-square (χ^2^) test using SAS 9.4 software.

In addition, we compared the allele frequencies of the c.495_496insCTCCC indel polymorphism of the *SPRN* gene between Italian goats^20^ and Korean native black goats (Fig. 2). The c.495_496insCTCCC indel polymorphism is well known for a relationship with scrapie susceptibility in Italian goats, where a significant difference in the allele frequency of c.495_496insCTCCC was found between scrapie-affected goats and healthy goats (P = 0.002)^20^. This result suggests that the c.495_496insCTCCC allele is associated with scrapie susceptibility. Interestingly, the allele frequency of the c.495_496insCTCCC indel polymorphism in Korean native black goats was significantly lower than in both heathy and scrapie-affected Italian goats (P < 0.001).

Furthermore, we estimated the impact of the nonsynonymous SNP c.416G > A (Arg139Gln) on the Sho protein using the PROVEAN program. PROVEAN predicted that R139Q was ‘Neutral’ with a score of −0.297 (Table 4).

**Table 4:** PROVEAN analysis of *SPRN* nonsynonymous SNP in Korean native black goats.

Variant	PROVEAN score	Prediction (cutoff = −2.5)
Arg139Gln	−0.297	Neutral

## Discussion

In this study, we investigated, for the first time, caprine *SPRN* polymorphisms in 213 Korean native black goats and found a total of 6 polymorphisms of the *SPRN* gene, including one novel nonsynonymous SNP, R139Q. We were also the first to annotate R139Q as ‘Neutral’ using PROVEAN. Finally, we compared the allele distribution of the c.495_496insCTCCC indel polymorphism between Korean native black goats and scrapie-infected and healthy Italian goats and identified significant differences among them in the allele frequencies of this indel polymorphism.

The prion gene family consists of four members, the *PRNP* gene, *prion-like* protein gene (*PRND*), *prion-related* protein gene (*PRNT*) and *SPRN* gene^30–36^. The former three genes are located on chromosome 13 and a strong genetic LD among these genes has been shown in previous studies^36–38^. In addition, the scrapie susceptibility-related *PRNP* SNPs showed a strong LD with *PRND* and *PRNT* SNPs. These results suggest that the SNPs of the *PRND* and *PRNT* genes are associated with scrapie susceptibility. Until now, the causal effect of the *PRND* and *PRNT* genes on scrapie susceptibility was unclear because this effect could be induced by an LD block. Another member of the prion gene family, the *SPRN* gene, is located on chromosome 26. Since the *SPRN* gene is located on a different chromosome from the *PRNP* gene, previous association studies for scrapie susceptibility of the *SPRN* gene were clearly not the result of an LD block with the *PRNP* gene. This indicates that the *SPRN* gene may be another potent candidate gene associated with the susceptibility to prion diseases regardless of the genotype of the *PRNP* gene.

The Sho protein encoded by the *SPRN* gene has a neuroprotective function similar to that of the prion protein and is expressed primarily in the neurons of the central nervous system (CNS). Sho is a glycosylphosphatidylinositol (GPI) anchor protein similar to the prion protein and shows high homology to the prion protein^39,40^. Previous studies have reported that the *PRNP* codon region 108–126 of the prion protein, including the palindromic hydrophobic core sequence, “AGAAAAGA”, which contributes to the conversion to the deleterious form of prion protein, interacts with the *SPRN* codon region 61–77 of the Sho protein, including “AGAAAGA”, which shows high homology with the prion protein^41^. In addition, the Sho protein affects the folding pathway of the prion protein. In a previous study based on a cell culture model, the PrP^Sc^ conversion rate increased in a dose-dependent manner with the amount of the Sho protein^42^.

Since the Sho protein shows prion disease-related functions, we estimated the impact of R139Q on the Sho protein using PROVEAN (Table 4). Although PROVEAN has predicted R139Q to be “Neutral”, a direct measurement of its impact on the function of the Sho protein is needed in the future.

In addition, polymorphisms in the 3′UTR of the *SPRN* gene can modulate the expression level of its mRNA^43,44^. In Italian goats, the allele distribution of c.495_496insCTCCC was significantly different between scrapie-affected goats and healthy goats, and the c.495_496insCTCCC allele seemed to impact scrapie susceptibility^20^. To verify the latter effect, an investigation of the mRNA expression level of the *SPRN* gene and a measurement of its impact on the prion infection rate are needed in the future. Notably, allele distribution of c.495_496insCTCCC in Korean native black goats is significantly different from that of scrapie-affected Italian goats. However, the allele distribution of c.495_496insCTCCC is also significantly different between healthy Italian goats and Korean native black goats (Fig. 2). This result could be due to the different origins of the two breeds; further investigation of c.495_496insCTCCC of the *SPRN* gene in various breeds is needed in the future.

There were differences in the number of goats used in each group, including healthy Korean, healthy Italian and scrapie-affected Italian goats. If the sample selection was biased, a small sample size could have affected the significance of the results. However, in the Italian goats, the author performed sample collection using a herd-stratified random-sampling method. In addition, the samples from the Korean native black goats were collected 5 times on 8 farms to avoid biased sample collection. Nevertheless, our sample size is somewhat limited. Thus, a case-control study in sample size-matched large groups to verify the association of the *SPRN* gene with susceptibility to prion diseases is highly desirable in the future.

To avoid biased results due to the prevailing effect of *PRNP* polymorphisms, the *PRNP* gene was analyzed and normalized based on *PRNP* polymorphisms. Italian goats showed extremely low allele frequencies of 146S, 146D, 154H and 211Q and goats with the 222K allele were excluded from analysis in a previous study^20,45^. Thus, we performed genotyping of 183 Koran native black goats and excluded 93 goats with 146S, 146D, 154H, 211Q and 222K for normalizing the analysis. Interestingly, this result was very similar to that of the original results shown in Fig. 2 (Supplementary Fig. 1).

In conclusion, we identified a total of 6 polymorphisms, including one nonsynonymous SNP in Korean native black goats, and analyzed the impact of nonsynonymous SNPs on the Sho protein. In addition, we evaluated the *SPRN* genetic type of Korean native black goats, which showed significantly different distributions of a scrapie-related indel polymorphism compared to Italian goats. To the best of our knowledge, this is the first genetic study of *SPRN* polymorphisms in Korean native black goats.

## Methods

### Ethical statement

All experimental procedures were approved by the Chonbuk National University Institutional Animal Care and Use Committee (IACUC number: CBNU 2017-0076). All experiments using Korean native black goats were performed in accordance with the Korea Experimental Animal Protection Act.

### Genomic DNA extraction

Blood samples were collected from 213 Korean native black goats at a slaughterhouse in the Republic of Korea. These samples were provided from 8 farms that are located in Jeollanam-do and were collected on five occasions from March 2016 to June 2016. The sample size used in the present study is large enough to identify rare polymorphisms, including below 1% genotype frequency^46^. In addition, the sample size is representative of the total population of Korean native black goats with a 95% confidence level and a confidence interval of 7. Whole blood treated with ethylenediaminetetraacetic acid (EDTA) was frozen at −80 °C prior to analysis. Genomic DNA was purified from 200 μl whole blood using the QIAamp DNA Blood Mini Kit (Qiagen, Valencia, California, USA) following the supplier’s instructions.

### Polymerase chain reaction (PCR) and DNA sequencing

To amplify the caprine *SPRN* gene, PCR was performed with gene-specific primers: Goat *SPRN*-Forward (5′-CCGTCCTCACAGAAGCTGAG-3′) and Goat *SPRN*-Reverse (5′-TCCTCAAGTCCTTCAGTCCCTG-3′). The primers were designed based on the genomic sequence of the caprine *SPRN* gene registered at GenBank (GQ267530.1). The PCR mixture contained of 10 μM each primer, 2.5 μl of 10× *Taq* DNA polymerase reaction buffer containing 25 mM MgCl_2_, 2.5 mM of each dNTP mixture and 2.5 units of DiaStar^TM^ *EF Taq* DNA polymerase (SolGent, Daejeon, Republic of Korea). The PCR was carried out as follows: predenaturation at 98 °C for 2 min; 33 cycles of denaturation at 98 °C for 20 sec, annealing at 56 °C for 30 sec, and extension at 72 °C for 1 min; and final extension at 72 °C for 5 min. The purification of PCR products for sequencing analysis was performed with a QIAquick Gel extraction Kit (Qiagen, Valencia, California, USA). The PCR products were directly sequenced with an ABI 3730XL sequencer (Applied Biosystems, Foster City, California, USA). Sequencing results were read by finch TV Version 1.4.0, and genotype analysis was performed.

### Statistical analysis

Analysis of  LD and haplotype distributions in Korean native black goats were performed using Haploview software (version 4.2, https://www.broadinstitute.org/haploview/haploview). Distributional differences in (HWE), HWE,  genotype, allele and haplotype were tested by Chi-square tests (χ^2^) and Fisher’s exact test using SAS 9.4 Software (SAS Institute Inc., Cary, North Carolina, USA).

### Evaluation of nonsynonymous SNP of the caprine *SPRN*  gene

We evaluated nonsynonymous SNPs found in the ORF with the PROVEAN program (http://provean.jcvi.org/index.php) to predict the biological impact of protein function and structure. The PROVEAN program performs a protein BLAST search by entering protein sequences and amino acid variations to identify homologous sequences and generate scores. A score below −2.5 is considered “deleterious”, and a score above −2.5 is considered “Neutral”.

## Supplementary information

Supplementary Figure 1
