# Supplementary material for: Scrapie susceptibility-associated indel polymorphism of shadow of prion protein gene (SPRN) in Korean native black goats
Source: Sci Rep. 2019 Oct 24;9:15261. doi: 10.1038/s41598-019-51625-8 (PMC6813300; doi:10.1038/s41598-019-51625-8)
Supplement: Supplementary file 1 — Supplementary Figure 1 [file 41598_2019_51625_MOESM1_ESM.pdf]

Scrapie susceptibility-associated indel polymorphism of shadow of prion protein gene (*SPRN*) in Korean native black goats

Yong-Chan Kim[1, 2, 3], Seon-Kwan Kim[1, 2, 3], Byung-Hoon Jeong[1, 2 *]

[1] Korea Zoonosis Research Institute, Chonbuk National University, Iksan, 54531, Republic of Korea

[2] Department of Bioactive Material Sciences and Institute for Molecular Biology and Genetics, Chonbuk National University, Jeonju, 54896, Republic of Korea

*** Corresponding author:**

Byung-Hoon Jeong, Ph.D.

Korea Zoonosis Research Institute, Chonbuk National University,

820-120, Hana-ro, Iksan, Jeonbuk 54531, Republic of Korea.

TEL: 82-63-900-4040, FAX: 82-63-900-4012, E-mail: bhjeong@jbnu.ac.kr

[3] These authors contributed equally to this work.

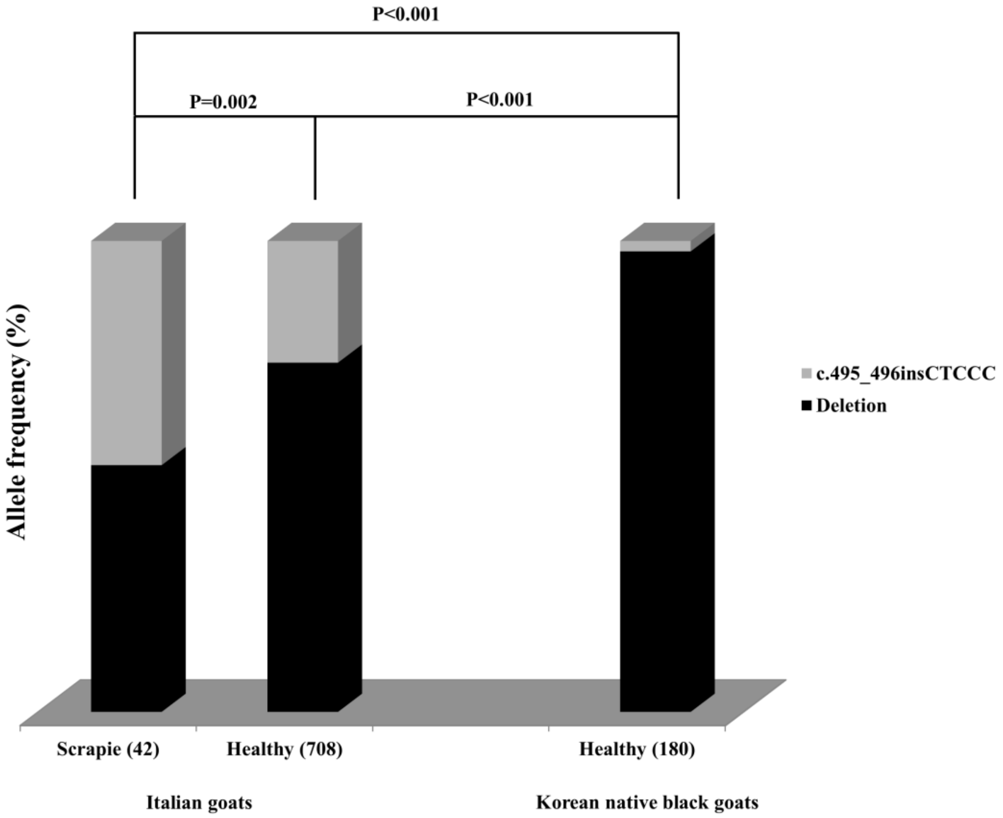
P<0.001
P=0.002
P<0.001
Allele frequency (%)
c.495_496insCTCCC
Deletion
Scrapie (42)
Healthy (708)
Healthy (180)
Italian goats
Korean native black goats

Supplementary Figure 1 Comparison of the allele distribution of the *SPRN* c.495_496insCTCCC indel polymorphism between scrapie-affected Italian goats and Korean native black goats normalized based on polymorphisms of the prion protein gene (*PRNP*). Korean native black goats with 146S, 146D, 154H, 211Q and 222K were excluded for normalizing analysis. The information regarding Italian goats was taken from a previous study [20]. The Italian goats were Maltese, Red Mediterranean, Capra dei Nebrodi and crossed breeds. Differences in allele distribution were calculated by the Chi-square ($\chi 2$) test using SAS 9.4 software.
